# Increased Prevalence of Mutant Allele *Pfdhps* 437G and *Pfdhfr* Triple Mutation in *Plasmodium falciparum* Isolates from a Rural Area of Gabon, Three Years after the Change of Malaria Treatment Policy

**DOI:** 10.1155/2016/9694372

**Published:** 2016-04-17

**Authors:** Jacques-Mari Ndong Ngomo, Denise Patricia Mawili-Mboumba, Noé Patrick M'Bondoukwe, Rosalie Nikiéma Ndong Ella, Marielle Karine Bouyou Akotet

**Affiliations:** Department of Parasitology-Mycology and Tropical Medicine, Faculty of Medicine, Université des Sciences de la Santé, BP 4009, Libreville, Gabon

## Abstract

In Gabon, sulfadoxine-pyrimethamine (SP) is recommended for intermittent preventive treatment during pregnancy (IPTp-SP) and for uncomplicated malaria treatment through ACTs drug.* P. falciparum* strains resistant to SP are frequent in areas where this drug is highly used and is associated with the occurrence of mutations on* Plasmodium falciparum* dihydrofolate reductase (*Pfdhfr*) and dihydropteroate synthetase (*Pfdhps*) genes. The aim of the study was to compare the proportion of mutations on* Pfdhfr* and* Pfdhps* genes in isolates collected at Oyem in northern Gabon, in 2005 at the time of IPTp-SP introduction and three years later. Point mutations were analyzed by nested PCR-RFLP method. Among 91 isolates, more than 90% carried* Pfdhfr* 108N and* Pfdhfr* 59R alleles. Frequencies of* Pfdhfr* 51I (98%) and* Pfdhps* 437G (67.7%) mutant alleles were higher in 2008. Mutations at codons 164, 540, and 581 were not detected. The proportion of the triple* Pfdhfr* mutation and quadruple mutation including A437G was high: 91.9% in 2008 and 64.8% in 2008, respectively. The present study highlights an elevated frequency of* Pfdhfr* and* Pfdhps* mutant alleles, although quintuple mutations were not found in north Gabon. These data suggest the need of a continuous monitoring of SP resistance in Gabon.

## 1. Introduction

In the early 1980s, sulfadoxine-pyrimethamine (SP) has been adopted for treatment of malaria cases when chloroquine treatment failed in many Sub-Saharan African countries [1]. However, soon after its introduction, resistance to SP has gradually emerged and spread widely from Asia to Africa [2, 3]. Nevertheless, SP is currently recommended by the World Health Organization (WHO) as intermittent preventive treatment for malaria in pregnant woman (IPTp) and children (IPTi) in malaria-endemic areas; it is also used as partner molecule of artemisinin derivatives [4, 5]. It is known that the presence of specific mutations on* Plasmodium falciparum dihydrofolate reductase* (*Pfdhfr*) and* Plasmodium falciparum dihydropteroate synthetase *(*Pfdhps*) genes encoding for proteins involved in the folate biosynthesis pathway of* P. falciparum* is related to SP resistance [6–8]. Molecular studies document the prevalence of these mutations in parasite populations across the African continent [9]. The* Pfdhfr* double mutations N51I-S108N and C59R-S108N confer intermediate levels of resistance while triple mutation N51I-C59R-S108N increases the parasite strains level of resistance in Africa [1]. The quadruple mutant N51I-C59R-S108N-I164R shows highest* ex vivo* resistance to pyrimethamine so far [6]. Similarly, an amino acid change at codon 437 on* Pfdhps *enzyme is due to the key mutation associated with sulfadoxine resistance. Additional changes at positions 540, 581, and 613 appear to increase its level [10]. The presence of quintuple mutation, composed of the combination of the* Pfdhfr* triple mutation (N51I-C59R-S108N) and the* Pfdhps* double mutation (437–540 or 437–581), increases the risk of therapeutic failure to SP [11]. However, the complexities of the evolutionary pressures that lead to the evolution of drug resistance are not well understood. Microbial systems that allow heterologous expression of malarial proteins provide the way to investigate patterns of evolution that can inform on the more complex factors that influence the evolution of drug resistance in clinical settings [12]. Indeed, preventive therapy based on SP use protects against parasite infection but induces the genesis of gametocytes and can promote the spread of resistant parasites [13]. In Gabon, IPTp-SP for pregnant women and artesunate-SP for uncomplicated malaria treatment are recommended by Malaria National Control Programme (MNCP) for malaria prevention and treatment, respectively. Five years after its introduction, the coverage with IPTp-SP two doses reached 60% [14]. Moreover, SP is still widely sold in pharmacies and distributors in remote areas from the country, despite the introduction of artemisinin combinations therapy (ACTs) for uncomplicated malaria treatment.

At the beginning of the year 2000 before IPT-p-SP implementation, previous studies describing the distribution of drug resistance molecular markers (*dhfr* and* dhps* genes) have been carried out in different areas of the country. In the southeast area, in the Haut-Ogooué Province, at Franceville, the proportion of isolates carrying a mutation at codon 108 was of 52%, while mutations at codons 51 and 59 as well as triple mutation were not frequent [15]. In contrast, at the same period and near Franceville, in Bakoumba, the triple mutant DHFR genotype was frequently detected (71.8%) whereas 64.3% combined at least three DHFR and one DHPS mutations [16].

In other regions, such as in the north of Gabon, at Oyem, similar investigations were not performed. In this area, regardless of the implementation of new strategies for malaria control, malaria prevalence remains high [17]. At the time of the study period, malaria prevalence tends to decrease; however, it was above 40% up to now. In this town surrounded by the forest and with a low level of urbanization, malaria transmission is perennial and children are frequently infected and constitute the main reservoir of parasites. Among many factors, the presence of resistant parasites could contribute to maintaining malaria burden in this region. Use of genetic information, for the early detection of resistance and monitoring of drug resistant malaria, is a helpful epidemiological tool. Indeed, molecular markers of resistance have emerged as epidemiologic tools to investigate antimalarial drug resistance even before becoming clinically evident. In this area, a previous study reported a high prevalence of* pfcrt* and* pfmdr1* drug resistance molecular markers associated with amodiaquine resistance, a partner molecule of artemisinin derivatives in ACTs [18]. This is the first study that reports baseline information on the characteristics and implications of antimalarial drug resistance, in the north of Gabon, with the aim to provide a data baseline on drug resistance. Thus, the prevalence of mutations on* Pfdhfr* and* Pfdhps* genes was compared in isolates collected in 2005 at the time of IPTp-SP introduction and three years later at Oyem in north of Gabon.

## 2. Methods

### 2.1. Study Site

Participants have been recruited through two different cross sectional studies carried out in 2005 and in 2008 at the regional hospital of Oyem (Centre Hospitalier Regional d'Oyem (CHRO)). The hospital of Oyem is the main hospital of the north of the country, a regional hospital. The majority of the population of the Oyem city which accounts for more than 35000 inhabitants and that of the surrounding villages receive healthcare in this hospital. CHRO is one of the five sentinel sites selected for malaria survey by the Malaria National Control Programme (MNCP). At Oyem, malaria prevalence remains above 40% among febrile children for a decade, and infection is predominantly caused by* P. falciparum* [17].

### 2.2. Patients and Samples

Isolates were collected from febrile children aged less than 11 years [19]. Patients who had positive blood smears and* P. falciparum* monoinfection were selected. The oral consent of the parents or legal guardians was also obtained. Blood was spotted on filter paper for further genetic analysis. Demographic data and medical history were reported.

### 2.3. Malaria Diagnosis

*P. falciparum* infection diagnosis was done by microscopic examination according to Lambaréné's method [20]. Smears were read using a light microscope with the 100x objective. Parasitaemia was expressed as a number of parasites per microliter of blood and parasite species was identified in the matched thin blood smears. Smears were considered as negative if the examination read under 100 of oil immersion fields did not reveal any parasites. Malaria case was defined as a patient with an axillary temperature >37.5°C and* Plasmodium falciparum* infection without WHO criteria of severity.

### 2.4. *Pfdhfr* and* Pfdhps* Genes Typing

Parasite nucleic acids were extracted from filter paper using QIAamp DNA Mini Kit (Qiagen, Hilden, Germany) according to the manufacturer's instructions and subsequently stored at −20°C until use.

*Pfdhfr* codons 108, 51, 59, and 164 and* Pfdhps* codons 437, 540, and 581 were analyzed using nested PCR-restriction fragment length polymorphism (PCR-RFLP) method. Mutations were detected by digesting PCR products with the restriction enzymes as described by Duraising et al. [21]. Amplicons and digested products were subjected to electrophoresis on 1.5 and 3.0% agarose gels, respectively, and visualized under UV transillumination light after staining. Digested products were compared with reference and local* P. falciparum* strains, which are 3D7: 51A-59C-108S-164I; PA2: 51A-59C-108A; C5: 51I-59R-108N-437A; and C4: 51N-59C-108N-437G; and for mutation at codons 540 and 581 local* P. falciparum* isolates. Samples containing both wild type and mutant alleles were classified as mixed infections.

### 2.5. Ethical Considerations

Department of Parasitology and Mycology (DPM) is committed by the Gabonese Ministry of Health represented by MNCP to carry out malaria diagnosis and antimalarial drug resistance monitoring throughout the country. Parents and legal guardians were informed about the studies and the consecutive molecular analysis. Their oral consent was obtained prior to inclusion in the study and before sample collection. Each patient with malaria positive blood smears was treated according to national recommendations at the time of the study.

### 2.6. Data Analysis

All data were entered and cleaned using Epi-info version 3.3.2. Analysis was performed with the Statview 5.0 software. All variables were compared using *χ* ^2^ test or Fisher's exact test. *p* value < 0.05 was considered statistically significant.

## 3. Results

### 3.1. Characteristics of Patients

Ninety-one* P. falciparum* isolates available, 29 from 2005 and 62 from 2008, were analyzed. Children median age was of 33  [31–37] months and their median parasite density of 53514 [31251–75776] p/*µ*L. Globally, 58% (*n* = 17) of the patients were male in 2005, while in 2008 the proportion of females (50%; *n* = 31) and males (50%; *n* = 31) was similar.

### 3.2. Prevalence of* Pfdhfr* and* Pfdhps* Mutations

*Pfdhfr* mutant alleles, N108 and R59, were found in 97.8% and 96.7% of the isolates, respectively. The frequency of I51 mutant allele was lower (80.2%, *n* = 73) although not statistically different (*p* > 0.05). No mutation was found at codons 540 and 581 of* dhps* gene, but 58% of the isolates carried the mutant allele at codon 437. Between 2005 and 2008, the proportions of mutant alleles N108 and R59 were comparable (Table 1). In contrast, the frequency of mutation at codon 51 was almost twofold higher in isolates from 2008: 95.2% versus 48.3% in 2005 (*p* < 0.01). The frequency of the double mutation 59-108 did not vary during the study period while the triple* dhfr* 108-59-51 mutation rate increased in 2008 reaching 91.9% (*n* = 57). As found for the* dhfr* I51 mutant allele, the proportion of isolates carrying the mutant allele 437G was almost twofold higher in 2008: 67.7% (*n* = 42) versus 37.9% (*n* = 11) in 2005 (*p* < 0.01) (Table 2). All parasites harbored a wild type allele at codons 164, 540, and 581 whatever the study period 2005 or 2008.

### 3.3. Haplotypes* Pfdhfr/Pfdhps*

For the calculation of the haplotype frequencies, samples with both* dhfr* and* dhps* genes analysis have been included. The frequency of haplotype combining the triple* Pfdhfr* mutation and the wild type allele at codon 437on* dhps* gene [108N-59R-51I-A437 (NRIA)] decreased in 2008: 29.9% versus 51.7% in 2005 (0.04). Inversely, parasites carrying the quadruple mutation (triple dhfr-437dhps mutation) were 1.9-fold more frequent in 2008 (Table 2). No quintuple (dhfr 51I/59R/108N and dhps 437G/ 540E) mutation was found.

## 4. Discussion

Occurrence and expansion of* P. falciparum* mutant genotypes frequency may depend on various factors such as year and location of study and age and clinical status of sampled population. In the present study, the prevalence of mutations on* Pfdhfr* and* Pfdhps* genes was compared in isolates collected in 2005 at the time of IPTp-SP introduction and three years later at Oyem, in the north of Gabon. Malaria prevalence there is above 40% and the treatment failures rates after SP administration were of 11.6% among children aged less than 5 years in 2005 [19]. In this area, the proportion of mutant alleles N108 and R59 of* Pfdhfr* gene in* P. falciparum* isolates was already high in 2005 and did not vary in 2008. Frequencies of these mutants' alleles were comparable to those found in isolates collected during the same period, between 2005 and 2006, at Lambaréné in the centre of Gabon and Libreville, the capital city of Gabon, two areas where malaria prevalence is different [22, 23]. Likewise, during the year 2006, in Kenya, more than 95% of* P. falciparum* isolates carried* dhfr* mutant alleles, a proportion that was already around 80% in the 1990s [24]. In contrast, in Iran and Senegal where malaria transmission is lower, during the same period, such high prevalence of* dhfr* mutant alleles was not found (<90%) [25, 26]. Concerning the mutant allele 51I, its frequency was above 75% in Lambaréné (79%) and Libreville (92%) while at Oyem it did not reach 50% (48.3%) [22, 23]. However, very quickly, in 2008, this allele was found in more than 95% of* P*.* falciparum* isolates collected at Oyem, a proportion comparable to those reported in regions from Congo (88%) and Benin (>90%) [27, 28]. Mutation at codon 164, which is associated with an elevated level of pyrimethamine resistance, was not detected. This mutation is rare in Africa [28, 29]. The triple* dhfr* mutation was also frequently detected at Oyem in 2008 (>90%), in a proportion comparable to the one found in Senegal (93%) and Benin (91.8%) in 2011 but higher compared to the ones found in Burkina Faso (54.3%) and Rwanda (78%) during the same period [26, 30]. In Iran, where malaria transmission is low and although sulfadoxine/pyrimethamine-artesunate combination has been adopted and recommended as first-line drug treatment the proportion of the triple mutation 108N-59R-437G is constant around 39% [25]. The combination of the triple mutation* Pfdhfr* and 437G mutation of the gene* Pfdhps* was found in two-thirds of the isolates analyzed in the present study, while in Senegal 44% of the isolates carried this haplotype [26].

The data obtained during the present study and those from previous investigations underline a different distribution and evolution of SP molecular markers resistance in Gabon. Indeed, while proportions of triple* dhfr* mutation and I51 mutant allele were already high in Lambaréné and Libreville during the years 2005-2006, at Oyem comparable frequencies were reached three years after in 2008. Nevertheless, between 2005 and 2011 at Libreville, the triple* Pfdhfr* mutation frequency increased from 92.9% to 100% and multiple mutation from 17.9% to 75.6% [31].* Dhps* mutant alleles at codons 581 and 540 as well as the* dhfr-dhps* quintuple mutation were not found at Oyem while they were detected at Libreville and Lambaréné. The presence of the* dhfr-dhps* quintuple mutation in* P. falciparum* isolates is reported to be a good indicator of the abandonment of the SP [9, 23, 32, 33]. These mutations were detected in 4% and 22% of the isolates from Lambaréné and Libreville, respectively. The main limitation of the present study could be the small number of isolates available and analyzed. However, these data provide important information allowing the setup of a database on antimalarial drug resistance molecular markers in Gabon. Indeed, although the number of samples is small, it is possible to draw from the present analyses accurate data and information on the prevalence of mutant parasites circulating in this area as performed by others [34]. These data already represent an “alarm” in this area where malaria transmission is high and suggest at least an increasing drug pressure in this area, related presumably to SP or other antifolates use.

## 5. Conclusion

The present study shows that* Pfdhfr* and* Pfdhps* mutant allele's frequencies are elevated, probably due to an increased use of sulfadoxine-pyrimethamine at Oyem. Three years after the adoption of WHO recommendation, frequencies of the combination of triple* Pfdhfr* mutation and* Pfdhps* 437G allele have risen, although among multiple mutations detected the quintuple mutation was not found. The monitoring of drug resistance molecular markers should be performed regularly and associated with the assessment of SP efficacy* in vivo* or* ex vivo* assays for Oyem.

## Figures and Tables

**Table 1:** Prevalence of mutations conferring resistance to sulfadoxine/pyrimethamine in *Plasmodium falciparum* isolates from Gabon.

Gene	Genotypes	2005		2008		*p*
		*n*	%	*n*	%	
*Pfdhfr*	108N	29/29	100	60/62	96.7	NS
	59R	27/29	93.1	61/62	98.4	NS
	51I	14/29	48.3	59/62	95.2	<0.01
	Double mutation: 51I-59R	21/29	72.4	62/62	100	<0.01
	Double mutation: 51I-108N	21/29	72.4	60/62	97.7	<0.01
	Double mutation: 59R-108N	29/29	100	60/62	96.7	<0.01
	Triple mutation: 51I-59R-108N	21/29	72.4	57/62	91.9	0.03

*Pfdhps*	437G	11/29	37.9	42/62	67.7	<0.01

**Table 2:** Prevalence of *Pfdhfr/Pfdhps* haplotypes.

Haplotypes: *Pfdhfr/Pfdhps*	2005		2008		*p*
	*n*	%	*n*	%	
108N-59C/R^*∗*^-51I-437G (NC/RIG)	0/29	0	2/55	3.6	NS
108N-59R-51I-437A/G^*∗*^ (NRIA/G)	1/29	3.4	0/55	0	NS
108N-59R-51I-A437 (NRIA)	15/29	51.7	16/55	29.9	0.04
108S-59R-51I-437G (SRIG)	0/29	0	1/55	1.8	NS
Quadruple mutation: 108N-59R-51I-437G	10/29	34.4	35/55	64.8	0.01

^*∗*^Parasites carrying mixed infections designated by two genotypes at the codon indicated above. NS: not significant. For the calculation of the haplotype frequencies, samples with both *dhfr* and *dhps* genes analysis have been selected.
